# Electronic and transport properties in circular graphene structures with a pentagonal disclination

**DOI:** 10.1186/1556-276X-8-258

**Published:** 2013-05-29

**Authors:** Esther Jódar, Antonio Pérez–Garrido, Fernando Rojas

**Affiliations:** 1 Departamento de Física Aplicada, Antiguo Hospital de Marina, Campus Muralla del Mar, UPCT, Cartagena, Murcia 30202, Spain; 2 Departamento de Física Teórica, Centro de Nanociencias y Nanotecnologías, UNAM, Apdo. 356, Ensenada, Baja California 22830, Mexico

**Keywords:** Graphene; Transport; Defects, 72.80.Vp, 72.15.Rn, 73.20.At

## Abstract

We investigate the electronic and transport properties of circular graphene structures (quantum dots) that include a pentagonal defect. In our calculations, we employ a tight-binding model determining total and local density of states, transmission function and participation number. For the closed structure, we observe that the effect of the defect is concentrated mainly on energies near to zero, which is characteristic of edge states in graphene. The density of states and transmission functions for small energies show several peaks associated with the presence of quasi-bound states generated by the defect and localized edge states produced by both the circular boundaries of the finite lattice and induced by the presence of the pentagonal defect. These results have been checked by calculating the participation number, which is obtained from the eigenstates. We observe changes in the available quasi-bound states due to the defect and the creation of new peaks in the transmission function.

## Background

The theoretical and experimental study of properties of graphene has attracted the attention of many authors in the last few years since a method to isolate single graphene layers was developed (the authors Geim and Novoselov were awarded with the Nobel prize). These graphene sheets may be stable enough to be freely suspended [1], which allows us to use them in solid state experiments. Besides, the electronic properties of graphene are surprising: one finds new quasi-particles described by the Dirac equation at low energies that behave like massless particles. This opens the possibility to study quantum electrodynamics properties in solid-state devices and to carry out new developments, e. g., biosensors (see other studies [2-10]).

**Figure 1:**
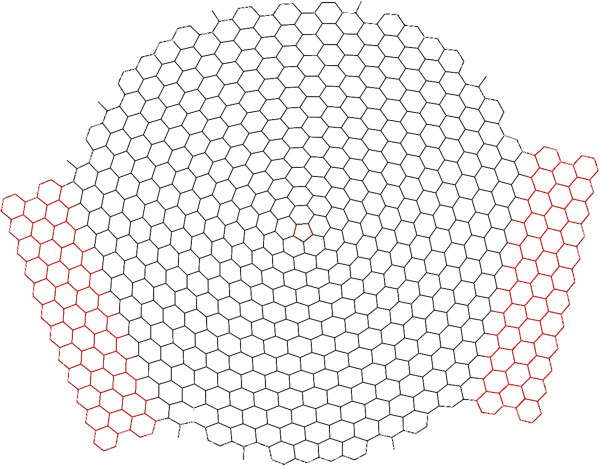
**Graphene sheet with the topological defect.** Schematic geometry of the graphene sheet studied in this work. Note the pentagonal defect placed at its centre (in red colour). This structure is connected to two semi-infinite graphene leads, which are partially shown in the figure (red colour).

The influence of defects and edges in graphene properties has been widely studied [11-13]. Other authors made similar studies to ours but considered different geometries: Zhang et al. [14,15] worked on transport with narrow ballistic ribbon of graphene with zigzag edges including topological defects. Carpio et al. [12] studied the electronic properties in a similar geometry but with dislocations consisting of heptagon-pentagon pairs in an hexagon lattice. The aim of this article is to study the combined effects, on the electronic properties, of the geometric structure and the presence of topological defects. We then consider a model with a pentagonal defect (disclination), henceforth PD, at the centre of a graphene sheet with a circular shape (see Figure 1). We characterize the electronic and transport properties with the local and total density of states, participation number and transmission function. This work can be useful for the search of structures suitable for confinement of Dirac electrons, which are the basis for the construction of nanoelectronic devices with graphene.

## Methods

Our geometry consists of a finite circular graphene quantum dot with 1,011 carbon atoms. For electronic transport, the quantum dot is connected to two semi-infinite leads. In Figure 1, we show the quantum dot and, partially, the semi-infinite leads. We employ a tight-binding model that only takes into account one *π*-orbital per atom. The overlap energy between nearest neighbours is taken as *t*=2.66 eV, where second-neighbour interactions are neglected. The advantage of using a single-band *π*-orbital model resides in its simplicity, being the general features of electronic transport in very good agreement with those obtained by more sophisticated approaches. The hamiltonian can then be written as

(1) $$Ĥ=-t\underset{<\text{ij}>\sigma }{\sum }(c_{i}^{‡}c_{j}+\text{hc}),$$

where $c_{i}^{‡}/c_{i}$ are the creation/annihilation operators of an electron in site *i*. We expand the wave function in terms of the site base. $|\Psi^{k}\rangle =\underset{i}{\sum }a_{i}^{k}|i\rangle$, where $a_{i}^{k}$ is the amplitude probability that the electron is to be in site *i* for the eigenstate *k*. We need to solve $Ĥ|\Psi^{k}\rangle =E^{k}|\Psi^{k}\rangle$. Four quantities are calculated to characterize the nature of the electronic and transport properties on two-circled structures, with PD and defect-free (ND) structures: the total density of states *N*(*E*), the local density of states *ρ*(*i*,*E*), the participation number *P*(*E*) and the transmission function *T*(*E*).

### Electronic properties for the closed system

The density of states is determined from the energy spectrum as

(2) $$N(E)=\frac{1}{D}\overset{D}{\underset{k=1}{\sum }}\delta (E-E_{k}).$$

Another useful property is the local density of states:

(3) $$\rho (i,E)=\frac{1}{D}\overset{D}{\underset{k=1}{\sum }}|\langle i|\Psi_{k}\rangle {|}^{2}\delta (E-E_{k}),$$

which measures how each site *i* contributes to the complete spectrum. For a fixed *E*, it characterizes the spatial nature of the state: it is localized when only few sites contribute to that energy, or extended when more sites participate. Finally, the participation number is defined as [16]

(4) $$P(E)=\frac{1}{\overset{D}{\underset{k=1}{\sum }}|\langle \Psi^{k}|\Psi^{k}\rangle {|}^{4}}.$$

It assesses the wave function spreading so it can help to find out the localized or extended nature of an electronic state. For a completely localized wave function *Ψ*^*k*^(*i*) is approximately *δ*_*ki*_→*P*≈1 while for a typical delocalized wave function on *D* atoms, *Ψ*^*k*^(*i*) is approximately $\frac{1}{\sqrt{D}}$, and then *P*≈*D*.

### Electronic transport properties: Green’s function method

We calculate transport properties for our graphene structures such as the density of states and the transmission function using Green’s function method. In order to obtain Green’s function, we use the following expression [17]:

(5) $$Ĝ(E)={\left(\mathrm{EÎ}-Ĥ-{\hat{\Sigma }}_{L}-{\hat{\Sigma }}_{R}\right)}^{-1},$$

where ${\hat{\sum }}_{L}$ and ${\hat{\sum }}_{R}$ are the self-energy terms of left and right leads, respectively, and Ĥ is the Hamiltonian of the conductor, i.e., in our case, the circular graphene sheet plus a few unit cells of the leads. In our approach, the contact leads at opposite sides of the circular graphene sheet is the graphene sheet itself extended to make the leads semi-infinite. This is equivalent to have reflectionless contacts in macroscopic conductors. Self-energy terms are calculated using the prescription ${\hat{\Sigma }}_{R/L}\left(i,j\right)=t^{2}{Ĝ}_{R/L}\left(k,l\right)$, where ${Ĝ}_{R/L}\left(k,l\right)$ is Green’s function of the semi-infinite lead (right or left) evaluated on sites k and l, which are in contact with sites i and j in the circular graphene sheet.

We only need to calculate ${Ĝ}_{R/L}$ in the sites in contact with the conductor. To do that, we use the formalism developed by López Sancho et al. [18]. This method has the advantage that the number of iterations close to singularities is very low compared to other transfer matrix methods, so it converges very fast and has been applied to graphene layers by other authors (see e.g. [19]). In this scheme, Green’s function is ${Ĝ}_{R/L}={\left(\mathrm{EÎ}-{Ĥ}_{00}-{Ĥ}_{01}\hat{T}\right)}^{-1}$, where ${Ĥ}_{00}$ is the Hamiltonian of one isolated graphene cell in the lead, and ${Ĥ}_{01}$ is the matrix that takes into account the interaction between two consecutive cells. For the calculation of *T*, we use the iterative method described in [18].

From Green’s function of the graphene structure, we calculate the transmission function and the density of states as [17]

(6) $$T(E)=\text{Tr}\left[{\hat{\Gamma }}_{L/R}{Ĝ}^{R}{\hat{\Gamma }}_{R/L}{Ĝ}^{A}\right]$$

(7) $$N(E)=-\frac{1}{\pi }\text{Tr}\left(\text{Im}Ĝ\right).$$

In Equation 6, *G*^R/A^ are the retarded and advanced Green’s functions, respectively, and ${\hat{\Gamma }}_{L/R}=i[{\hat{\sum }}_{L/R}^{R}-{\hat{\sum }}_{L/R}^{A}]$. We denote the trace of the matrix considered by “Tr”, which is extended over the whole matrix.

## Results and discussion

**Figure 2:**
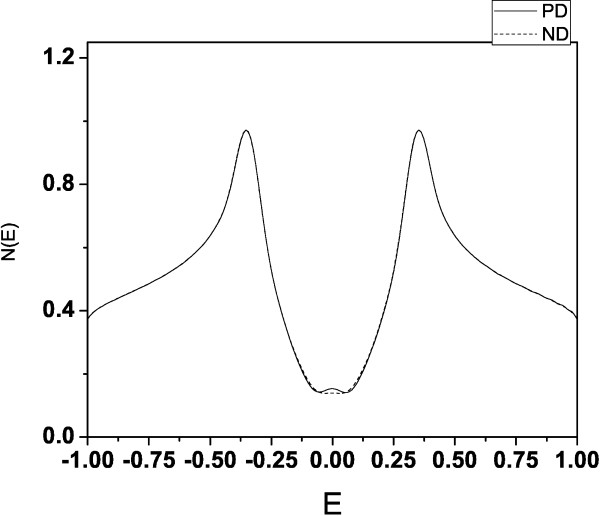
**Density of states for the closed structure.** Total density of states for the structure with no defects (dashed line) and with a pentagonal defect (continuous line). Note the non-null density near zero as a manifestation of the edge defects.

**Figure 3:**
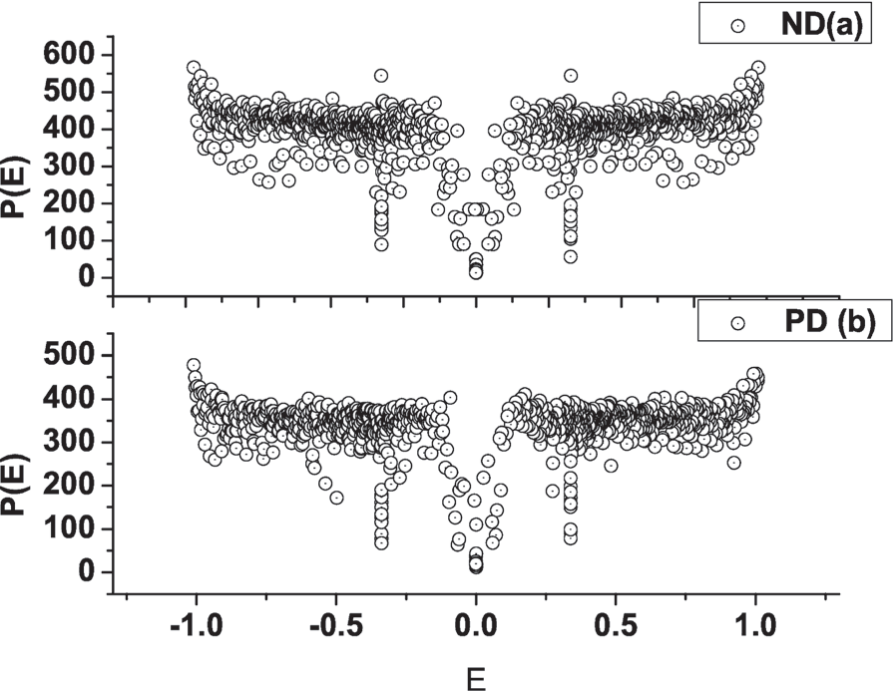
**Participation number for the closed structure.** Participation number *P*(*E*) of the available energy states for the structure with no defects (**a**) and with the pentagonal defect in the centre (**b**).

We have obtained different properties of graphene structures with and without pentagonal defects, in order to evaluate the influence of the defect and the geometry on their electronic properties. For the closed structure, we have calculated the total density of states, which is shown in Figure 2, for both the defect-free structure (dashed line) and with PD (continuous line). We see that the density for the structure with PD shows a shoulder near *E*=0, indicating the existence of additional edge states induced by the presence of the PD and the circular shape of the structure. The behaviour of the participation number confirmes these findings (see Figure 3a for the ND and Figure 3b for the PD structures). One can observe that *P*_PD_<*P*_ND_, which suggests that the PD induces localized states beyond the known edge states in the structure with ND. In addition, near *E*=0, the structure of the participation number is slightly different in both structures. This shows that the nature and the number of the edge states are different, which can be seen in the local density function for this energy.

**Figure 4:**
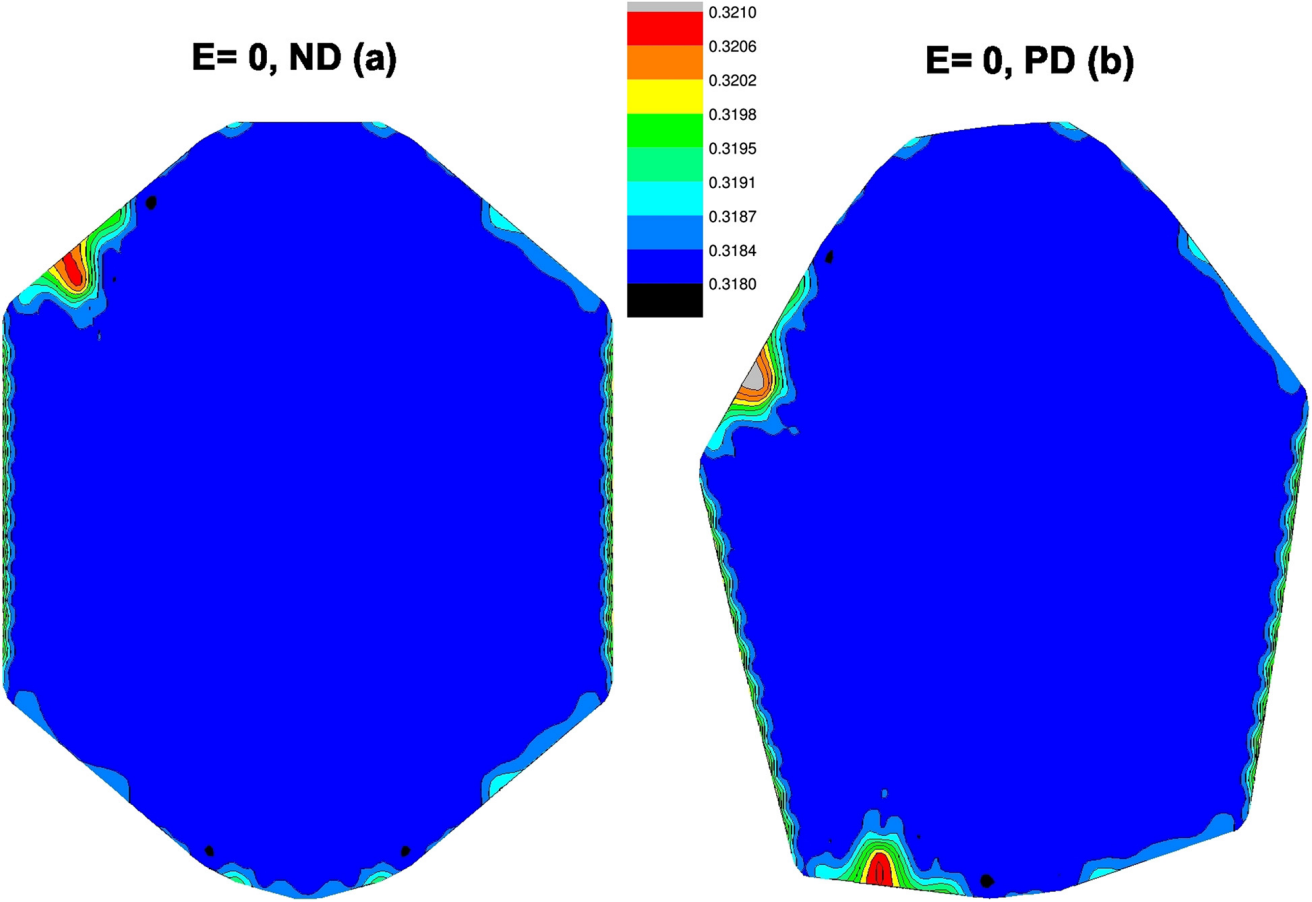
**Local density of states for *E* = 0.** Spatial distribution of the local density for *ρ*(*i*,*E*) for the energy *E* close to zero in (**a**) a structure with no defect and (**b**) one with the pentagonal defect in the centre. Due to single-bond atoms (see Figure 1), the quantum dot is not fully symmetric around a central vertical axis.

**Figure 5:**
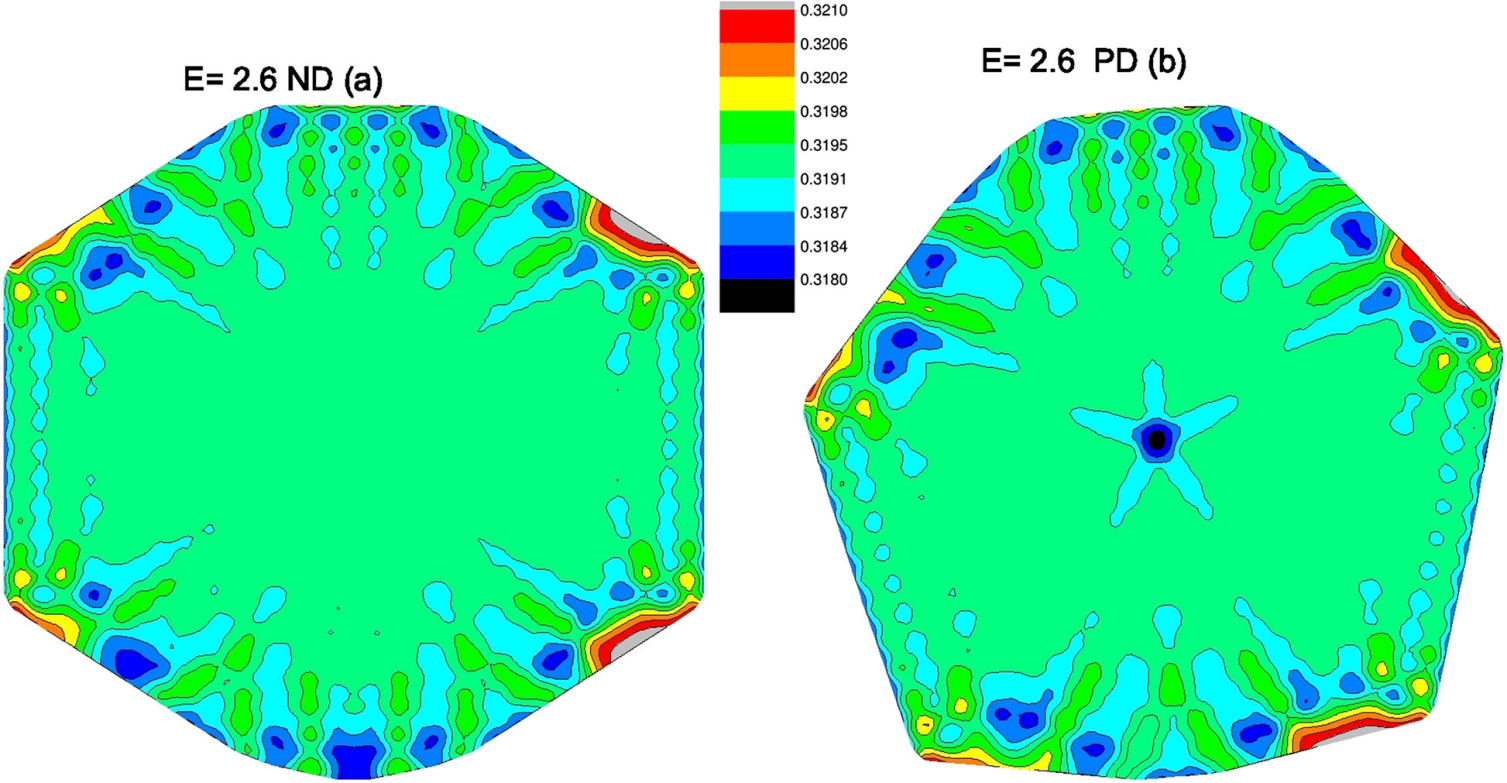
**Local density of states for *E* = 2.6 eV.** Same as Figure 6 but for the energy *E* = 2.6 eV.

The edge states are localized so only few states contribute to a certain site; this is shown in Figure 4 for the local density of states at *E*=0 and *ρ*(*i*,*E*=0) for both the ND (Figure 4a) and PD (Figure 4b). Clearly, these are edge states, and the PD structure shows contribution from two zones, compared to the ND structure with one. The effect of the PD on the density of states near *E*=0 is of geometrical nature; the whole structure is affected by the presence of the pentagon since it changes the relative orientation of the edge sites and induces the creation of edge states. This has to do mainly with the atom rearrangement in the lower part of the structure, which creates new edge states and, clearly, the PD sites do not have an explicit contribution to such sites. For larger values of *E*, in the local density of *ρ*(*i*,*E*=2.6), more sites contribute to that energy (see Figure 5). Specifically, we see the contribution of sites around the PD as it can be seen in Figure 5b, where a star shape appears. The rest of the sites contribute more or less similarly to the structure with ND (Figure 5a).

**Figure 6:**
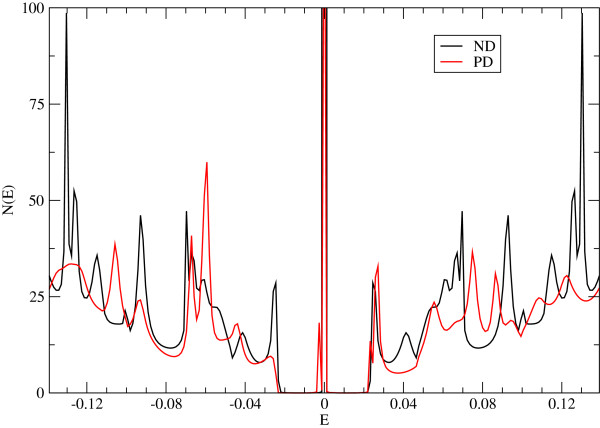
**Density of states for the open structure.** Density of states for the graphene sheet with the pentagon at its centre (red line) and without it (black line). Note the displacement of the different peaks.

**Figure 7:**
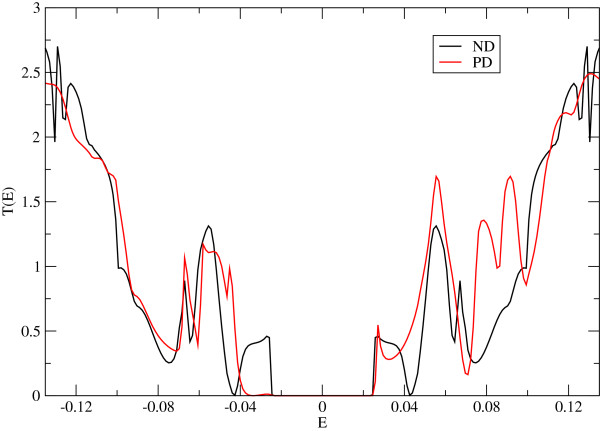
**Transmission function for the open structure.** Transmission function with the pentagon at its centre (red line) and without it (black line). Note the additional peaks for the case with the defect.

As the change in behaviour with the presence of PD is near zero energy (around the Fermi energy), we concentrate in the analysis of the transport properties around such energy. We have also checked our previous results in the open structure calculating the density of states (Figure 6) and the transmission function (Figure 7). The density of states shows several peaks associated with both the presence of quasi-bound states (due to the circular confinement and the defect) and localized edge states due to circular boundaries of the finite lattice. These results are clearly observed in the peak structure of the transmission function (Figure 7), where we observe changes in the quasi-bound states available to transport and the creation of new peaks in the transmission function.

## Conclusion

We have investigated the electronic and transport properties of circular graphene layers with a pentagonal disclination. In particular, using a tight-binding model, we have calculated the density of states, transmission function, participation number and local density of states of the structure with and without defects. The density of states for the structure with the PD shows several peaks that are associated with new localized states, which have been checked by calculating the local density of states and the participation number. We observe changes in the available quasi-bound states due to the defect and new peaks of the transmission function. Comparing these results, we conclude that there are more quasi-bound states in the structure with the defect, states associated with both the presence of quasi-bound states related to the atoms belonging to the defect and others due to the circular confinement and edge states due to circular boundaries of the finite lattice and the defect.

## Abbreviations

ND: Defect-free structure; PD: Pentagonal defect.

## Competing interests

The authors declare that they have no competing interests.

## Authors’ contributions

The work presented here was carried out in collaboration among all authors. FR defined the research theme. EJ carried out the calculations under APG’s supervision. All of them have discussed the results and wrote the manuscript. All authors read and approved the final manuscript.
